# Innovation Centers in Health Care Delivery Systems: Structures for Success

**DOI:** 10.2196/33961

**Published:** 2022-02-10

**Authors:** Onil Bhattacharyya, Justin Shapiro, Eric C Schneider

**Affiliations:** 1 Women’s College Research Institute Women's College Hospital Toronto, ON Canada; 2 Department of Family and Community Medicine University of Toronto Toronto, ON Canada; 3 Department of Family Medicine Women's College Hospital Toronto, ON Canada; 4 Institute of Health Policy, Management and Evaluation University of Toronto Toronto, ON Canada; 5 Temerty Faculty of Medicine University of Toronto Toronto, ON Canada; 6 Department of Health Policy and Management T H Chan School of Public Health Harvard University Cambridge, MA United States; 7 The Commonwealth Fund New York, NY United States

**Keywords:** innovation, digital health, value-based care, quality improvement, delivery science, value, structure, success, health care delivery, reform, survey, outcome, experience, strategy

## Abstract

The need to support innovation in health care delivery was prompted by payment reforms and access to digital tools and has been accelerated by the shift to virtual care as part of the COVID-19 pandemic response. Prior to the pandemic, a growing number of health systems set up innovation centers to focus on creating new services and exploring new business models relevant to value-based care. This is distinct from process improvement or implementation science, and often needs a different set of incentives to succeed within a large organization. We used a national survey to identify a diverse sample of innovation centers, and interviewed leaders to describe their aims, organizational structures, and activities. They all aim to improve patient outcomes and experience while reducing costs, but their strategic focus may differ. The centers also vary in their reporting structure, how they build internal capacity, and how they measure success. We highlight the range of strategies through examples of projects that improve quality, reduce costs, and generate new revenue. While the optimal forms and impact of innovation centers are still emerging, the fiscal pressures and the rapid uptake of digital technologies present opportunities for the redesign of health services in the postpandemic era. The experiences of these centers illustrate a set of approaches to increase any organization’s capacity for innovation.

## Introduction

While improvement and innovation are ubiquitous terms, the COVID-19 pandemic response has made innovation more of a core requirement than a marketing tool in health care. Both aim to solve problems, improve outcomes, and reduce costs, but there are key distinctions. Improvement is an iterative and incremental process. It involves testing and measuring the effects of small changes to optimize the reliability of services [1]. Fundamentally, improvement enhances a system by removing perceived defects and evaluating the consequences.

Innovation, by contrast, takes a less incremental approach. Innovation is about creating or adapting novel ideas that may disrupt the status quo or solve a specific problem. It is particularly useful when the end goal is vaguely defined, a new service of unclear value is developed, or the environment and underlying needs are changing rapidly [2]. Health systems face growing challenges in controlling costs while providing patient-centered care. The current model of care is being overwhelmed by an aging population with increasingly complex and chronic diseases. Productivity in the US health care system dropped by 0.8% per year between 1990 and 2007 [3]. Even before the COVID-19 pandemic, these conditions created an environment ripe for disruption, and innovation has become an indispensable approach to solving health care’s most daunting challenges.

The health care sector proved its resilience and flexibility by adapting to novel and rapidly evolving conditions as the pandemic unfolded [4]. However, as we enter the postpandemic era, health care organizations must enhance their capacity for innovation to meet pressing medical, social, and fiscal demands [4].

## The Rise of Innovation Centers

Within the last 10 years, a growing number of health systems have set up innovation centers [5], with at least 110 in the United States [6,7]. Motivated by payment reforms, the potential for new revenue streams, changing patient expectations, and the prospect of losing out to competitors, health system leaders are betting that innovation centers will enable them to offer more patient-centered, affordable care. Their focus is often on creating new services that may be unrelated to current care processes. This is distinct from classic quality improvement [8] and broader than delivery science [9] since it also involves exploring new business models [10]. Business model innovation can include the design of new services, but it also considers modifying internal cost structures or revenue sources, as well as partnerships and delivery channels [11]. Since they focus on generating and eliminating novel options, and some strategies may undermine the dominant business model, innovation centers require a different set of skills and incentives to succeed within a large organization [12]. While the idea of concentrating innovation efforts in one center has been challenged [13], this model is becoming more common and is likely a feasible starting point [14]. Looking to innovation methods that have galvanized change in other industries like user-centered design, agile development, and novel digital tools, they aim to create new scalable health services [12]. Value-based payment reforms by the US federal government and other payers are creating incentives to experiment with ways to deliver care that are independent of current processes.

Innovation centers are new enough that health system leaders are still learning how to build and manage them [15]. This novelty is yielding a variety of approaches as shown by a prior Commonwealth Fund survey of innovation centers and by several case studies [6,16,17]. We examined the responses from 33 innovation centers to identify centers that appeared to have developed and spread an innovative model of care at the time, and found 10, to which we added another 3 through snowball sampling. This paper enriches the existing literature by further elucidating the link between the goals of various innovation centers and the structures built by organizations to fulfill those goals. While the internal capabilities of health systems in the discipline of service design and business model innovation are where quality improvement was two decades ago, we feel that these skills are essential to the development of sustainable, person-centered health systems.

## The Aims and Organization of Innovation Centers

Nearly all centers share the aim of improving patient health status and patient experience while reducing costs. However, in practice, their strategic focus differs. Centers may focus on quality improvement, streamlining back-end processes, internal cost reduction, or revenue generation. These areas of focus suggest different structures, with examples of centers reporting to leaders in clinical quality, information technology (IT), or finance. Some emphasize improving clinical quality and operations within the organization, while others seek to create scalable models of care or new technologies with the hope of generating revenue externally. For example, the Virginia Mason Institute (hereafter referred to as Virginia Mason) focuses almost entirely on improving clinical quality within the organization (based on an adaptation of the Toyota Production System). The center is based within their quality improvement department, and the center’s leadership reports to the senior vice president of quality and safety. NewYork-Presbyterian Innovation Center also focuses on internal operations, but its innovation center is distinct because it is based within the IT department and its staff report to the chief information officer. A team of IT experts and managers support system-wide implementation of mature mobile technologies to improve access to care and reduce costs. In 2019, the Hauser Institute for Health Innovation was launched to focus specifically on advancing telemedicine capabilities.

The University of Pittsburgh Medical Center (UPMC) Enterprises emphasizes the objectives of reducing costs and increasing revenue and is co-led by the treasurer of the health system. It includes a venture fund that seeds investments in start-up companies with products or services that can both support UPMC’s internal operations and generate revenue from returns on investments. Northwell Ventures is primarily focused on venture investing to generate revenue. It is a distinct division within the health system that consists of an investment fund and a team of finance and management experts that work with internal and external partners to develop new technologies or models of care, but scale them up internally only if they identify interested outside investors, signaling an external market and a higher likelihood of financial return. While all of these examples aim to fulfill the broad goal of improving quality through innovation, their strategies can be delineated in practice according to their focus, structures, and key performance indicators.

## Staffing and Expertise to Support an Innovation Center

Modern approaches to innovation seek a deep understanding of the user experience along with rapid prototyping and iterative testing [5]. Health systems have not traditionally employed individuals with expertise in user-centered design or entrepreneurial management methods such as lean startup. Health systems must choose between training current staff, hiring staff with new skills, or partnering with external groups. Efforts to build internal capacity include engaging current staff through internal grants, crowdsourcing for new ideas, asking employees to vote on potential strategies to adopt, and training staff in new methods. In some cases, innovation centers create new leadership positions, such as chief innovation officer, and hire new types of staff, such as designers, developers, entrepreneurs, or finance experts. Some build facilities for rapid prototyping, offsite clinics that serve as test environments, and some have tried to build new information systems infrastructure that connects data from health systems, patients, and software and application developers [18]. Some centers hire new types of staff who work in separate divisions, while others are embedded within frontline care provision. For example, UPMC Enterprises employs engineers, designers, and clinicians who have dedicated time to develop new technologies and models of care. By contrast, Virginia Mason has employed staff to train clinical and administrative staff throughout the larger organization in methods like user-centered design while assisting them as they redesign clinical services while engaged in routine service delivery. Some groups partner externally to acquire new skills, like the Cedars-Sinai Accelerator, a collaboration between the Cedars Sinai-Medical Center and Techstars, which engaged health system leaders to identify priority areas and select start-ups to address them in cohorts with US $100,000 in financial support and mentoring to launch in a clinical environment. Others have engaged design firms and included them on advisory boards, such as the Mayo Clinic Center for Innovation engaging with the firm IDEO.

## Strategic Decisions About Structuring Innovation Efforts

Many health systems displayed remarkable agility as they rapidly shifted in-person services to virtual platforms during the pandemic to reduce in-person care, but can they build this into a core capability postpandemic? There are several questions health system leaders should consider as they decide on the best structure and focus for their innovation efforts. First, is the primary goal to improve core business or develop new businesses and service lines? Second, what relevant expertise and competencies already exist in-house? These assets could include software developers, designers, technology transfer offices, or connections with early-stage entrepreneurial companies. What level of risk is the organization willing to tolerate? Those willing to tolerate higher risk may wish to go beyond improving current core activities to take on higher-risk, higher-reward options. Those with lower risk tolerance may wish to stay closer to improving core functions and focus on innovations that protect existing business from the changing landscape. What form should organizational investment in the innovation center take? Options range from smaller investments, such as partnerships and internal training of current staff, to larger investments in seed funding for a center that is expected to become financially independent, to ongoing core funding to build new facilities and hire new types of staff. Lastly, can these goals be achieved within an existing department, or do they require a new, dedicated center?

## What Have Innovation Centers Achieved to Date?

There are few formal evaluations of the results of innovation center activities. As health care organizations are just beginning to learn what innovation centers can achieve, most have taken a flexible approach to evaluating them. For example, the Mayo Clinic Center for Innovation is often assigned strategy development projects, such as imagining the future of payment or quality improvement. These are tasks that other divisions are ill-equipped to pursue, and that may not have easily measurable short-term impacts. Nevertheless, there are successful examples of innovation centers improving quality, reducing costs, or generating revenue. On the quality improvement front, Virginia Mason developed and refined a clinical care protocol that reduced the time from onset of sepsis to start of treatment from 6 hours to 1 hour and refined the protocol to further reduce the time to 30 minutes, a success well beyond what other organizations have achieved. While this resembles quality improvement in approach (changes in the responsibilities of clinical staff), the magnitude of the improvement (12-fold reduction in time to treatment) is in line with what one expects from breakthrough innovations. This highlights the potential overlap between the outcomes of multiple small tests of change and radical new practices.

In the area of cost reduction, UPMC Enterprises provided seed funding to a start-up that refined natural language processing to improve coding accuracy, quadrupled coder productivity, and identified previously missed conditions to risk adjust patients for Medicare Advantage. In this instance, UPMC went from launching an untested technology developed by an external start-up to achieving system-level cost savings within one year. For revenue generation from new sources, UPMC Enterprises developed an internal consulting model for their transition to an accountable care organization. UPMC then turned the team and its protocol into an independent company called Evolent Health. From a US $38 million initial investment, the company had an initial public offering of over US $1 billion, and UPMC’s stake grew to US $300 million [19,20]. While this illustrative example showed benefit in a short time frame, the centers took many years to build this capacity and have engaged in many projects before having successes like this.

## How Have Innovation Centers Fared During the COVID-19 Pandemic?

Managing the response to the COVID-19 pandemic made innovation an organizational imperative, and innovation centers across the country responded in various ways. An overarching lesson from the COVID-19 response was that innovation rapidly became an organizational, rather than departmental, imperative. The task of innovating was no longer the realm of those in formal roles within innovation centers; innovation was on display as a core function for all health system leaders. Further, innovations largely focused on improving or maintaining access to services during a time of physical distancing (often through remote care), as opposed to focusing on improving quality.

UPMC Enterprises continued to invest heavily in health care innovations throughout the pandemic. UPMC Enterprises led the development of a digital patient portal for virtual appointment scheduling, expanded telemedicine capacity, and helped portfolio companies grow [21]. Butterfly Network, a medical imaging company in which UPMC Enterprises invested, went public through a US $1.5 billion acquisition deal [22]. Notably, the organization also announced a US $1 billion investment in the life sciences by 2024 [21].

Cedars-Sinai Medical Center continued to host its start-up accelerator program, albeit virtually [23]. Techstars ran a Global Startup Weekend, bringing together thousands of innovators globally to collaborate on creative solutions to the unique challenges posed by the COVID-19 pandemic [24]. The Hauser Center for Health Innovation continued to focus on innovations related to remote patient monitoring and increasing telemedicine capabilities more broadly [25].

Virginia Mason continued to improve internal quality and quickly adapted to shifting supply chain metrics and guidance from the US Centers for Disease Control and Prevention (CDC) [26]. The organization also accelerated its multiyear plan to increase telehealth capabilities [26].

## Conclusion

Innovation is a set of approaches, not a destination. While the optimal forms, goals, and impact of innovation centers are still emerging, the fiscal pressures of new payment models and the potential for new digital health technologies to challenge current delivery models are real. Most health care organizations remain focused on regulatory compliance, using quality improvement to make relatively small improvements to existing processes. But this may not yield the changes needed to thrive in the future. The early experiences of innovation centers illustrate the variety of paths available to those seeking to grow their organization’s capacity for innovation. As health systems adapt to the postpandemic era, it remains to be seen whether innovation centers will meet the growing medical, technological, and fiscal demands.

## Abbreviations

CDC: US Centers for Disease Control and Prevention
IT: information technology
UPMC: University of Pittsburgh Medical Center
